# Therapeutics and the Lesser Circulation

**Published:** 1894-12-01

**Authors:** Benjamin Ward Richardson


### Therapeutics and the Lesser Circulation.

By Sir Benjamin Ward Richardson, M.D., F.R.S.

### I.—PRELIMINARY CONSIDERATIONS.

Whilst writing the articles on "toxic neutralisations" my mind has been recalled to the subject of the lesser circulation—the course of blood through the lungs during diseased conditions, and the power we, in medicine, possess of regulating the blood current through this lesser circulation.

Nothing has ever impressed me more in the observation of the symptoms of diseases of serious nature—in which life is at stake—than the importance of practical knowledge of the method of governing the blood current. The fact, observed for ages past, that the arteries after death are commonly left empty of blood once caused them to be considered naturally as vessels containing air or spirits, and indicates how often death must occur as a final arrest of the circulation on the pulmonic side. The fact did not escape the acute notice of William Harvey, who observed that in our dissections we usually find a large quantity of blood in the veins, little in the arteries, much in the right ventricle of the heart, and little in the left, circumstances which probably led the ancients to believe that the arteries, as the name implies, contained nothing but spirits during the life of the animal, and from which he drew the inference "that the true cause of the difference is, perhaps, that as there is no passage to the arteries save through the lungs and heart, when an animal has ceased to breathe and the lungs to move, the blood in the pulmonary artery is prevented from passing into the pulmonary veins, and from thence into the left ventricle of the heart; but the heart not ceasing to act at the precise moment as the lungs, but surviving them and continuing to pulsate for a time, the left ventricle and arteries go on distributing their blood to the body at large, and sending it into the veins and receiving none from the lungs, are soon exhausted, and are left, as it were, empty." This is one of the observations of the discoverer of the circulation. But he has a second, which deals more strictly with the question before us in its relation to disease. "The emptiness of the arteries in the dead body, which probably misled Erasistratus in supposing that they (the arteries) only contained aerial spirits, is caused, he supposed, by this: "That when respiration ceases the lungs collapse and the passages through them are closed. The heart, however, continues for a time to contract upon the blood, whence we find the left auricle more contracted, and the corresponding ventricle as well as the arteries at large empty, because there is no supply of blood flowing round to fill them. In cases, however, in which the heart has ceased to pulsate, and the lungs to afford a passage to the blood simultaneously, as in those who have died from drowning, or syncope, or who die suddenly, the arteries, as well as the veins, are full of blood."

In these passages Harvey clearly, and almost correctly, puts before us the great clinical fact that death as a rule takes place by reason of failure in its course in the lesser circulation. The common people, who are often strangely correct in the views they take, seem to have the same idea. They always speak of the act of dying as "giving forth the last breath," for they conceive that breathing means life. Of course they are not altogether correct; they do not express what is, but what seems to be. We have to go a step further; we have to consider the proportions that should exist between the amount of blood that comes to the surface of the lungs to be aërated, and the amount of air that comes to such surface in a given period of time in order that oxidation of it may take place. We have also to consider the quality of air that enters to perform its duty, and the quality of blood that rises to be acted upon by the air. The air may not be in a condition for proper oxidation; the blood may not be in a condition to receive air, so that a true asphyxia may begin even in the blood; and, further than this, there may be such an imperfect regulation in regard to pressure of air and blood, that the balance of the continuity in the delicate lung structures, vesicular and capillary, may be destroyed with the certainty of death, usually, from the circumstance Harvey observed, that the left side of the heart does not receive its blood supply, but empties itself and its arteries without being again re-fed from the pulmonic veins.

The natural formula of this balance may be thus expressed. In a given period of time, say one minute, the right side of the heart must so regulate the blood pressure that there shall be a certain pressure of blood on the capillary surface of the lung as there is pressure of air on the vesicular surface. In like manner at the same time the thoracic mechanism must so regulate the air pressure in the vesicles that there shall be a proper pressure of air on the vesicular surface as there is of blood on the capillary surface. This is a mechanical necessity, as it occurs to my mind, and if it be true, the merest break in the balance required must have the most important bearings on life during its healthy condition, and still more during conditions of disease.

The balance required is regulated, not so much by the number of cardiac or thoracic movements, as by the force of the movements their balance and their equality. But for such provision every irregularity in the motion of the heart or of the thorax would be registered in the lung by lesion of structure. In the act of running this is well expressed. When a man commences to run rapidly the heart takes brief precedence of respiration in regard to relative activity or motion, and the sensation of breathlessness is the result. After a short time, if there be usually a good balance, the breathing movements come abreast of the cardiac, the breathlessness passes off, and the running is easily sustained until the force of combustion—the mainspring—fails. In short, for true disturbance of the balance on either the respirating or circulating side, there must be, necessarily, either direct mechanical obstruction or direct failure on one side.

In the treatment of disease, error of balance between the circulation and respiration, in one or other of the methods named, is daily offered for study. The grand question is whether we have any practical means at command for restoring correct balance when that is disturbed or broken.

